# Unprotected sexual practice and associated factors among People Living with HIV at Ante Retroviral Therapy clinics in Debrezeit Town, Ethiopia: a cross sectional study

**DOI:** 10.1186/1742-4755-11-56

**Published:** 2014-07-21

**Authors:** Etsub Engedashet, Alemayehu Worku, Gezahegn Tesfaye

**Affiliations:** 1 Management Science for Health, Monitoring and Evaluation Department, Addis Ababa, Ethiopia; 2 College of Health sciences, School of Public Health, Addis Ababa University, Addis Ababa, Ethiopia; 3 Department of Public Health, College of Health and Medical Sciences, Haramaya University, Harar, Ethiopia

**Keywords:** Unprotected sex, Factors, People living with HIV/AIDS

## Abstract

**Background:**

Magnitude of unprotected sexual practice among PLHIV is generally high in African countries including Ethiopia. Understanding the practice in Ethiopia could have public health significance. However little is known about the issue of unprotected sexual practice among PLHIV in Ethiopia. Hence, this study was aimed to assess unprotected sexual practice and associated factors among PLHIV at ART clinics in Debrezeit town.

**Method:**

Institution based cross-sectional study was conducted. A total of 667 PLHIV were included in the study. Systematic random sampling technique was used to select participants. Analyses were done using SPSS for windows version 15. A crude and adjusted odds ratio with 95% confidence interval was used to measure association between different factors and unprotected sex.

**Result:**

The prevalence of unprotected sexual practice among PLHIV was 22.2% [95% CI: (19.0-25.4)]. Factors associated with unprotected sexual practice include: being female (AOR = 2.1, 95% CI (1.1, 3.9)), being divorced/widowed/separated (AOR = 4.9, 95% CI (2.1, 11.6)), length of stay with the current partner for ≥ 49 months (AOR = 3.3, 95% CI (1.9, 5.7)) and not discussing or partly discussing about safe sex and condom use with sexual partner (AOR = 17.1, 95% CI (8.9, 32.8)).

**Conclusion:**

High proportions of PLHIV were found to engage in unprotected sex. Information Education and Communication (IEC) on safe sex for PLHIV should target females, those who stayed longer with their partner and divorced/widowed/separate ones.

## Background

The vast majority of people newly infected with Human Immune Virus (HIV) in sub-Saharan Africa are infected during unprotected heterosexual intercourse (including paid sex) and onward transmission of HIV to new born and breast fed babies. Having unprotected sex with multiple partners remains the greatest risk factor for acquisition of HIV in this region [1].

With expanded access to Ante Retroviral Therapy (ART), the growing number of people living longer with HIV forms a potential source of infection. Unless people living with HIV consistently practice safer behaviors, they can place themselves at risk of sexually transmitted infections, including other strains of HIV, and place others at risk for HIV infection [2].

Unprotected sex remains a concern among People Living with HIV (PLHIV). The magnitude of the problem varies from country to country. Generally the magnitude of unprotected sexual practice among PLHIV is very high in African countries ranging from 40.1% among males and 46.3% among females in Cape Town, South Africa to 83% in Uganda [3,4]. However, the magnitude of unprotected sexual practice among PLHIV in countries other than Africa is relatively low when compared to the African situation. It ranges from 23% in Croatia to 58% in Vietnam [5,6].

There is some evidence that heavy alcohol use which is more often seen in PLHIV than the general population may be a risk factor for unprotected sex. Advances in HIV treatment have created optimistic beliefs about HIV transmission risk and sexually transmitted infection vulnerability, which can influence motivation to practice safer sex [7,8].

A number of issues were raised regarding risk factors related to HIV infection. The fear of stigma and lack of belief in the reality of HIV, also occasionally led PLHIV to continue to engage in risky behavior including alcohol abuse and unprotected sex. Many people did not feel able to moderate their lifestyles, again for fear of having others discover that they were HIV-positive. In some cases, PLHIV continued to have unprotected sex with their partners, even though they were aware of the risk of infecting their partner, rather than begin using condoms, and have their partner discover their HIV-positive status [9].

According to the national fact sheet 2010 from Acquired Immune Deficiency Syndrome (AIDS) resource center of Ethiopia, the adult HIV prevalence is 2.4% and adult HIV incidence is 0.26 and total HIV positive population is 1,216,908 [10,11]. Having this much total PLHIV in the whole country can have serious impact on the general population. Additionally with the presence of ART people infected with HIV can live longer and if they don’t practice safe sex, they will put others at risk of new HIV infection.

Understanding the practice in Ethiopia could have public health significance there by generating and providing evidence based information for policy makers, program planners and health service providers on the problem to design and implement appropriate intervention mechanisms. However, little is known about the issue of unprotected sexual practice among PLHIV in Ethiopia in general and the study area in particular. A study done in Addis Ababa [12] found a high level of unprotected sex among PLHIV but the study only included hospital patients selected by consecutive approach. In this study we aimed to determine the magnitude and factors associated with unprotected sex among randomly selected PLHIV who have follow up ART clinics of a hospital and health center in Debrezeit town, Ethiopia.

## Methods and materials

### Study area and period

The study was conducted in Debrezeit town which is located south east of Addis Ababa, the capital city of Ethiopia. According to 2007 census report of Ethiopia, the total population of the town is 100,114 from which male constitutes 47938 (47.8%) and female constitutes 52176 (52.2%) [13]. Two health facilities (Bishoftu hospital and Bishoftu health center) which have ART service were included in the study. The hospital has a total client of 5,085 on chronic care of whom 2,266 were on ART and the health center has a total client of 617 on chronic care of whom 147 were on ART. The study was conducted from October to December, 2011.

### Study design

Institution based cross-sectional study was conducted.

### Source population

All PLHIVs who have follow up at ART clinics in the public health facilities of Debrezeit town.

### Study population

All PLHIVs who have follow up at ART clinics in the public health facilities of Debrezeit town. Those patients who were 18 year of age and above, were volunteer to participate in the study, had been sexually active in the past one year and have at least two clinic visits were included in the study. Those patients who cannot communicate properly, mentally ill or seriously ill were excluded from the study.

### Sample size determination

The sample size for the first objective was determined using the following assumptions: Z score at 95% CI =1.96, marginal error = 4%, and prevalence of unprotected sex among PLHIV 36.9% [12] yields a total sample size of 560 including 10% none respondent rate. The sample size for the second objective was determined using a study conducted in Vietnam where gender and marital status were found to be associated factor for unprotected sex [6]. A study done in Montgomery identified alcohol consumption as associated factor for unprotected sex [14]. Using open epi version 2.3, by taking 95% confidence interval, unexposed to exposed ratio (1:1), 80% power and 10% non-response rate reveals the following result. Gender (practice in unexposed 58% and practice in exposed 72.7% gives a total sample of389) and marital status (practice in unexposed 57.5% and practice in exposed 68.8% yields a total sample of 667) and alcohol consumption (practice in unexposed 43% and practice in exposed 57% yields a total sample of 466).

From the above sample size calculations the minimum larger sample size was 667. The total sample size was allocated to the health center and the hospital proportionally. Bishoftu hospital accounts for 89.17% of the total PLHIV and the health center accounts for 10.82% of the total PLHIV in the study area. Therefore the total sample for the hospital was 594 and for the health center 73.

### Sampling procedure

The study participants were selected using systematic random sampling technique. The sampling interval was determined based on two month patient flow in each health facility. The total number of PLHIV who came for follow up at ART clinic of Bishoftu hospital in two months were estimated to be 6000 making the sampling interval of 10. The total number of PLHIV who came for follow up at ART clinic of Bishoftu health center in two months were estimated to be 220 making the sampling interval of 3. In order to avoid double counting patients coming for follow up were asked if they were interviewed for this study in previous days and were excluded if they did so.

### Data collection

A structured questionnaire was used for collecting the data. The questionnaire was adopted from a previous study [12] by adding other questions related to the study objectives. It has questions to screen those who were sexually active, socio demographic characteristics, sexual practice before becoming positive, current sexual practice and substance use. The questionnaire was prepared first in English and translated to Amharic then back to English to check for consistency. Face to face interview was used to collect the data. The data collectors were two health officers who work at ART clinics in the health facilities.

### Study variables

The outcome variable was “Unprotected sexual practice”: any sexual intercourse done without using condom or where condom is not used consistently in the past one month. The independent variables were socio demographic characteristics, health status related factors, and partner related factors and substance use.

### Data analysis

The data was first cleaned manually and given a code then entered to a computer. EPI info version 3.5.1 was used for data entry and then it was exported to SPSS version 15.0 for further data analysis. Descriptive statistics was done for frequency distribution. Bivariate and multivariate analysis using logistic regression was done to see the association between dependent and independent variables. In the bivariate analysis crude odds ratio and p-value were calculated. Variables with p-value of < 0.2 were taken to multivariate model for further analysis.

### Data quality control

The data collectors and a supervisor were given one day training on the data collection tool. At the hospital data collectors were daily supervised by the principal investigator. Data collectors at the health center were supervised by a health officer on a daily basis. Prior to the actual data collection, the questionnaire was pre-tested on 20 participants. After pre-testing, difficult questions were revised and after adjustment of the questions the actual data collection was conducted.

### Ethical consideration

Ethical clearance was obtained from the Institutional Review Board (IRB) of University of Gondar. Additionally support letter was obtained from the Oromia Regional Health Bureau. To assure confidentiality data collectors were from the ART clinic staffs. Informed consent was obtained from each participant before conducting the interview. Participants were informed of the purposes, procedures, risks and benefits of the study. No name or personal identifying information was written on the questionnaire. The participants had the right not to participate in the research and their involuntariness would not pose any negative consequences on them as well as on the service they get from the health facility.

## Result

### Socio demographic characteristics

A total of 667 people living with HIV were included in this study and the response rate was 100%. Most of them 418 (62.7%) were females and 249 (37.3%) were males. The mean age of the participants is 34.13 ± 7.74 (SD) (Table 1).

**Table 1:** Socio demographic characteristics of PLHIV at ART clinics in public health facilities in Debre Zeit town, Ethiopia, October - December, 2011 (N = 667)

**Characteristics**	**Categories**	**Frequency**	**Percentage**
Sex	Male	249	37.3
	Female	418	62.7
Educational status	Unable to read and write	194	29.1
	Read and write	157	23.5
	Up to grade 8	117	17.5
	High school	163	24.4
	College/university	36	5.4
Age	18-28	177	22.6
	29-33	168	25.2
	34-39	171	25.6
	≥40	151	22.6
Religion	orthodox	554	83.1
	Muslim	16	2.4
	Protestant	94	14.1
	Catholic	1	0.1
	Wakefeta	2	0.3
Ethnicity	Oromo	386	57.9
	Amhara	215	32.2
	Tigre	24	3.6
	Guraghe	28	4.2
	Other	14	2.1
Current occupation	Government employee	56	8.4
	Private employee	149	22.3
	House wife	180	27
	Daily laborer	147	22
	Merchant	57	8.5
	Farmer	41	6.1
	Other	37	8.5
Average monthly income	≤300	108	26.7
	301-500	118	29.2
	501-812	77	19.1
	>812	101	25.0

### Duration of follow up, health status and sexual practice before being HIV positive

Considering the follow up of the study participants at ART clinic, majority of them 324(48.6%) have 13-48 months of follow up, 112 (16.8%) have follow up below 12 months and 231 (34.6%) have follow up above 48 months. Among the study participants 590 (88.5%) have CD4 count above 200 cells/mm^3^ while the rest have below 200 cells/mm^3^. Majority of the study participants 533(79.9%) are currently taking ART.

Among all the participants in the study nearly half of them were tested for HIV and knew their HIV status by PIHCT (51%). Length of time since becoming positive varies among the study participants as 84 (12.6%) of them have known their HIV status before 12 months, 304 (45.6%) before 13 -48 months and the rest know their HIV status before 48 months. The study participants had different marital status before becoming HIV positive. Majority 549 (82.3%) of them were married, 61 (9.1%) were single, 11 (1.6%) were divorced, 18 (2.7%) were separated and 28 (4.2%) were widowed.

Moreover, before becoming positive more than half of the study participants 452 (67.8%) had one sexual partner whereas 157 (23.5%) had two sexual partner and 58 (8.7%) had three or more sexual partner. Condom utilization varies before becoming positive. Most participants 557 (83.5%) did not use condom before becoming HIV positive. However about 110(16.5%) reported to use condom before becoming positive. Of the 110 (16.5%) participants, only 41 (37.3%) reported to use condom always whereas the rest did not use condom consistently.

### Current marital status, partner related characteristics, condom use and sexual desire

Currently 81 (87.1%) were married, 35 (5.2%) were single, 29 (4.3%) were separated, 11 (1.6%) were divorced, and 11 (1.6%) were widowed. Different sexual partnerships were reported by the study participants in this study. Among the study participants, 648 (97.2%) reported to have one sexual partner in the last one year. Of whom, 629 (94.3%) identified their partner as primary/steady partner and 19 (2.9%) as casual partner. Around 19 (2.8%) of the participants have reported sexual partnership with more than one partner. Among these only 3 (0.4%) have identified their partners as steady partner, 14 (2.1%) as a casual partner and 2 (0.3%) as having both type of partner. Regarding sexual desire after becoming HIV positive, around 65.5% reported to have decreased sexual desire whereas 33.6% reported to have the same sexual desire and only 0.9% reported to have sexual desire that has increased.

There was a different distribution of characteristics among males and females PLHIV regarding their partner related characteristics which was described in Table 2.

**Table 2:** Partner related characteristics and condom use among males and females PLHIV in public health facilities of Debre Zeit town, Ethiopia, October - December, 2011

**Characteristics**		**Male**		**Female**	
		**Number**	**Percentage**	**Number**	**Percentage**
Number of partner in the last one year	One	238	95.6%	410	98.1%
	More than one	11	4.4%	8	1.9%
Length of stay with current partner	≤12 months	76	32.8%	183	45.5%
	13-48 months	60	25.9%	87	21.6%
	≥49 months	92	41.4%	132	32.6%
Partner serostatus for those having one partner	Negative	65	27.3%	97	23.7%
	Positive	154	64.3%	251	61.2%
	Unknown	19	8.0%	62	15.1%
Partner sero-status for those having more than one partner and use condom	Negative	-	-	-	-
	Positive	3	30%	2	28.6%
	Unknown	7	70%	5	71.4%
Partner sero-status for those having more than one partners and don’t use condom	Negative	-	-	-	-
	Positive	-	-	1	100%
	Unknown	1	100%	-	-
Condom use	Yes	223	89.6%	353	84.4%
	No	26	10.4%	65	15.6%
Consistency of condom use	Always	208	93.3%	310	87.8%
	Almost always (more than half)	10	4.5%	17	4.8%
	Sometimes	1	0.4%	5	1.4%
	Almost never	4	1.8%	21	5.9%

**Figure 1:**
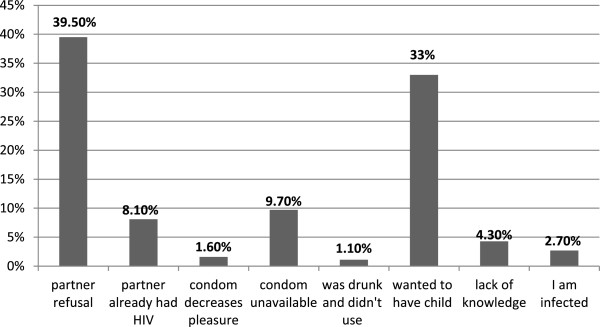
Reasons for not using condom consistently among PLHIV at ART clinics in public health facilities of DebreZeit town, Ethiopia, October - December, 2011.

Among the study participants 503 (81.9%) reported to use condom consistently whereas the rest do not use condom consistently. About 53 (7.9%) reported not to use condom at all in all their sexual intercourse. Among those individuals not using condom consistently and not using at all, 158 (96.3%) did not use condom when they have intercourse with a steady partner, 5 (3%) with casual partner and only 1 (0.6%) with both type of partner.Different reasons have been mentioned by the study participants for not using condom consistently (Figure 1).

### Magnitude of unprotected sex

**Figure 2:**
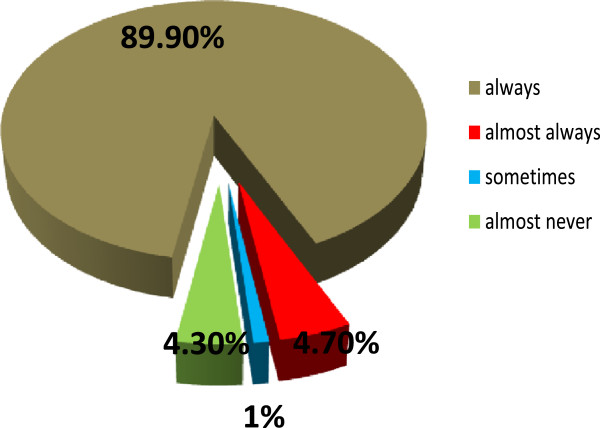
Consistency of condom use among PLHIV at ART clinics of public health facilities in DebreZeit town, Ethiopia, October - December, 2011.

Condom utilization in the last one month was reported by 86.4% of the study participants from whom about 518 (89.9%) uses condom consistently (Figure 2). Only 91 (13.6%) did not use condom at all during sexual intercourse in the last one month. The magnitude of unprotected sexual practice among PLHIV was 22.2% [95% CI: (19.0-25.4)]. Among the study participants 144 (22.2%) who had one sexual partner were found to engage in unprotected sex whereas only five individuals who had more than one partner were engaged in unprotected sex.

### Last sexual encounter, discussion on safe sex, condom use and sexual partner sero-status

In this study 89.4% of PLHIV reported to use condom during their last sexual encounter. Around 595 (89.2%) of the participants discuss with their sexual partner about condom utilization and safe sex whereas around 7.3% do not discuss at all and 3.4% discuss partly. Among the study participants with one sexual partner, about 162 (25%) have a sero-negative sexual partner, 405 (62.5%) have sero-positive sexual partner and the rest (12.5%) do not know the sero-status of their sexual partner.

Among those with multiple sexual partner and use condom, around 12 (70.6%) have a partner whose sero-status is unknown and 5 (29.4%) have sero-positive partner.

### HIV status disclosure and substance use

Most participants 94.6% have disclosed their sero-status to their sexual partner whereas the rest did not. Different reasons were mentioned by the participants for not disclosing their sero-status. Majority of them 14 (38.6%) reporting fear of stigma, 6 (16.7%) fear of divorce, 4 (11.1%) fear of discrimination and the rest mentions other reasons.

In this study about 144 (21.6%) reported to consume alcohol in the past one year from whom around 61 (42.4%) reported to consume alcohol once per week, 18 (12.5%) twice per week, 9 (6.3%) three times per week, 3 (2.1%) seven times per week and the rest reported to consume alcohol during holidays. Only 10 participants reported to chew khat and 4 participants to smoke cigarettes.

### Factors associated with unprotected sexual practice

Bivariate analysis was done to see the association of each factor with unprotected sex. Then variables that have p-value of <0.2 were included in the multivariate analysis to control for possible confounders. On the bivariate analysis different variables were found to be significantly associated with unprotected sex. These includes sex (p-value = 0.006), age ≥ 40 (p-value = 0.06), length of follow up at ART clinic (p-value = 0.000), length of time since becoming positive (p-value = 0.000), condom use before becoming positive (p-value = 0.032), current marital status (p-value = 0.000), length of stay with the current partner (p-value = 0.001), discussion about condom use and safe sex (p-value = 0.000) and disclosure (p-value = 0.000). Hence all the variables that meet the criteria were taken to multivariate analysis.

On multivariate analysis four variables becomes independently associated with unprotected sex. Those who were females (AOR = 2.1, 95% CI (1.1, 3.9)), divorced/separated/widowed (AOR = 4.9, 95% CI (2.1, 11.6)), have length of stay with the current partner for ≥ 49 months (AOR = 3.3, 95% CI (1.9, 5.7)) and who don’t discuss or partly discuss about safe sex and condom use with sexual partner (AOR = 17.1, 95% CI (8.9, 32.8)) were more likely to engage in unprotected sex (Tables 3 and 4).

**Table 3:** Socio demographic factors associated with unprotected sex among PLHIV in public health facilities of Debre Zeit town, Ethiopia, October - December, 2011

**Characteristics**		**Unprotected sexual practice**		**COR**	**AOR**
		**Yes**	**No**		
Sex	Male	41 (16.5%)	208 (83.5%)	1.00	1.00
	Female	107 (25.6%)	311 (74.4%)	1.75 (1.17,2.61)*	2.10 (1.14, 3.90)**
Age	18-28	48 (27.1%)	129 (72.9%)	1.00	1.00
	29-33	41 (24.4%)	127 (75.6%)	0.87 (0.54,1.41)	0.87 (0.47, 1.63)
	34-39	37 (21.6%)	134 (78.4%)	0.74 (0.45,1.21)	0.87 (0.47, 1.64)
	> = 40	22 (14.6%)	129 (85.4%)	0.46 (0.26,0.80)*	0.65 (0.29, 1.42)
Educational status	Unable to read and write	39 (20.1%)	155 (79.9%)	1.26 (0.49,3.23)	0.56 (0.10, 1.66)
	Read and write	31 (19.7%)	126 (80.3%)	1.23 (0.47,3.22)	0.68 (0.23, 2.01)
	Up to grade 8	37 (31.6%)	80 (68.4%)	2.31 (0.89,6.04)	1.13 (0.38, 3.36)
	High school	35 (21.5%)	128 (78.5%)	1.37 (0.52,3.55)	0.82 (0.28, 2.39)
	College/university	6 (16.7%)	30 (83.3%)	1.00	1.00
Current marital status	Married	111 (19.1%)	470 (80.9%)	1.00	1.00
	Single	11 (31.4%)	24 (68.6%)	1.94 (0.92,4.08)	0.92 (0.22, 3.81)
	Divorced/widowed/separated	26 (51%)	25 (49%)	4.40 (2.45,7.92)*	4.89 (2.07, 11.56)**

*Statistically significant before multivariate analysis.

**Statistically significant after multivariate analysis.

**Table 4:** Other factors that associated with unprotected sex among PLHIV in public health facilities of Debre Zeit town, October - December, 2011

**Characteristics**		**Unprotected sexual practice**		**COR**	**AOR**
		**Yes**	**No**		
Length of follow up at ART clinic	<=12 months	41 (36.6%)	71 (63.4%)	1.00	1.00
	13-48 years	65 (20.1%)	259 (79.9%)	0.44 (0.27,0.70)*	1.142 (0.39, 3.37)
	> = 49 months	42 (18.2%)	189 (81.8%)	0.39 (0.23,0.64)*	0.67 (0.23, 2.00)
CD 4 count	<=200	23 (29.9%)	54 (70.1%)	1.00	1.00
	>200	125 (21.2%)	465 (78.8%)	0.63 (0.37, 1.07)	0.92 (0.44, 1.94)
Length of time since being positive	<=12 months	34 (40.5%)	50 (59.5%)	1.00	1.00
	13-48 months	57 (18.8%)	247 (81.3%)	0.34 (0.20,0.57)*	0.35 (0.11, 1.20)
	> = 49 months	57 (20.4%)	222 (79.6%)	0.38 (0.22,0.64)*	0.80 (0.25, 2.58)
Condom use before being positive	Yes	33 (30%)	77 (70%)	1.00	1.00
	No	115 (20.6%)	442 (79.4%)	0.61 (0.39,0.96)*	0.71 (0.37, 1.35)
Length of stay with current partner	<=12 months	39 (15.1%)	220 (84.9%)	1.00	1.00
	13-48 months	33 (22.4%)	114 (77.6%)	1.63 (0.98,2.74)	1.78 (0.93, 3.42)
	> = 49 months	64 (28.1%)	164 (71.9%)	2.20 (1.41,3.44)*	3.26 (1.86, 5.71)**
Condom use and safe sex discussion	Yes	95 (16%)	500 (84%)	1.00	1.00
	No/partly	53 (26.4%)	19 (73.6%)	14.68 (8.32,25.91)*	17.11 (8.92, 32.81)**
Disclosure	Yes	129 (20.4%)	502 (79.6%)	1.00	1.00
	No	19 (52.8%)	17 (47.2%)	4.35 (2.20,8.61)*	2.16 (0.55, 8.45)
Alcohol consumption	Yes	39 (27.1)	105 (72.9%)	1.00	1.00
	No	109 (21%)	414 (79%)	0.71 (0.46, 1.08)	1.07 (0.60, 1.91)

*Statistically significant before multivariate analysis.

**Statistically significant after multivariate analysis.

## Discussion

In this study the magnitude of unprotected sexual practice among PLHIV was 22.2%. Being female, being divorced/widowed/separated, length of stay with the current partner for ≥ 49 months, and not discussing or partly discussing about safe sex and condom use with sexual partner were factors significantly associated with unprotected sex.

It was found that among PLHIV the magnitude of unprotected sex is 22.2% with 95% CI: (19.0%, 25.4%). The prevalence in this study is smaller when compared with a study conducted in informal settlement of Kenya (28%) and in Nigeria (56%) [15,16]. The difference with the Kenyan study might be due to the difference in study setting where this study was conducted in a slum area (Kibera) in which the participants have a relatively low economic and educational status. Thus low economic and educational status could tempt them to engage more in unprotected sex. On the other hand the difference with the Nigerian study might be due to the different in set up of the two study areas. Again the finding is smaller when compared with a study conducted in Addis Ababa [13]. The reason for this difference might be due to the relatively shorter duration of time (one month back sexual practice) used to measure the magnitude of unprotected sex in this study compared to the Addis Ababa study (three months back sexual practice) which might make the magnitude in this study smaller.

Moreover compared to an institution based cross sectional study conducted in (3 US metropolitan areas, a study conducted in Argentina and another study conducted in Atlanta [4,17,18], the prevalence of unprotected sex in this study is still smaller. The discrepancy might be due to the face to face interview method used for data collection as this might make the study participants to hide their actual practice. On the contrary the compared studies used self-administered questionnaires for collecting the data. However the finding is almost the same compared to another hospital based cross sectional study done in South west Ethiopia where the prevalence of unprotected sex was 24% and the same applies also when it is compared to a study conducted in Croatia where the magnitude was 23% [5,19].

Sex was significantly associated with unprotected sex where females are more likely to practice unprotected sex than males with (AOR = 2.12, (1.14, 3.91)). This might be due to the low economic and educational status of female participants compared to male participants. This finding is similar to a study conducted in Cape Town, South Africa which identified female gender to be significantly associated with unprotected sex. It is also the same when it is compared to a study conducted in the informal settlement of Kenya where females were more likely to engage in unprotected sex compared to males. Same applies when it is compared to a study conducted in India where being female was found to be unlikely to use condom than males [16,20,21].

It was also found that current marital status was significantly associated with unprotected sex. Those PLHIV who are divorced/widowed/separated are more likely to practice unprotected sex compared to those who are married (AOR = 4.90, (2.08, 11.58)). The fact that they are not married in which they don’t have a husband/wife might be contributing reason for their engagement in unprotected sex. This finding contradicts with a study conducted in Kenya and Malawi where those who were married or in cohabiting relation are less likely to use condom [22].

Moreover it was found that those who stayed with their partner for more than 49 months are about three times more likely to engage in unprotected sex than those who stayed for less than 12 months with (AOR = 3.21, (1.85, 5.57)). This might be due to the longer duration of stay. This is because the longer they stay together the trust that they will have on each other would be more and therefore they might tend to engage in unprotected sex.

Additionally those who don’t discuss or partly discuss about safe sex and condom use with their sexual partner are more likely to practice unprotected sex than those who discuss about condom use and safe sex with their sexual partner with (AOR = 17.03, (8.89, 32.63)). The reason might be due to the fact that discussing about condom use and safe sex would make both partners to avoid unprotected sex. Hence not to discuss or partly discuss can potentially make them to engage in unprotected sex. Compared to a cross sectional study conducted in Addis Ababa, the finding is the same where not to discuss about condom use and safe sex was an aggravating factor and independently associated with unprotected sex [13].

Lastly the programmatic implication of this study is that, the current effort to empower and educate women should be mainstreamed with the current HIV/AIDS programs targeting PLHIV. Educational messages targeting on PLHIV should consider the duration of stays of the couples and marital condition, and peer education programs should be designed to facilitate free discussion about sexual issue among HIV positive couples.

The strength of the study; this research has addressed an important area of public health which has an impact on prevention of further transmission of HIV/AIDS, HIV super infection and treatment failure and the study used primary data. This study also has some limitations. Due to the sensitive nature of sexuality and face to face interview social desirability bias may be introduced, which could underestimate the magnitude of unprotected sex. And those who don’t have follow up in a health facility and who do not come to the health facilities in the study period were excluded from the study which lead to selection bas and may limit generalization.

## Conclusion

Significant proportions of people living with HIV/AIDS were found to practice unprotected sex. The most common reason for not using condom consistently was that their partner/s does not want to use condom. Those participants who are female, separated/widowed/divorced, staying longer with a current partner and do not discuss about condom use and safe sex with their sexual partner were found to be more likely to engage in unprotected sex. Information Education and Communication (IEC) on safe sex for PLHIV should target particularly on females, those who stayed longer with their partner and those who are divorced/widowed/separate. Counseling should include educating patients on how to enhance discussion with sexual partner regarding condom use and safe sex.

## Abbreviations

ACIPH: Addis Continental Institute of Public Health; AIDS: Acquired immune deficiency syndrome; AOR: Adjusted odds ratio; ARC: AIDS Resource Center; ART: Ante retro viral therapy; CD 4: Cluster of differentiation 4; CI: Confidence interval; ETB: Ethiopian Birr; HAART: Highly active ante retro viral therapy; HIV: Human immunodeficiency virus; IRB: Institutional review board; OR: Odds ratio; AOR: Adjusted odds ratio; COR: Crude odds ratio; PLHIV: People living with HIV; PMTCT: Prevention of mother to child transmission of HIV/AIDS; SD: Standard deviation; SPSS: Statistical package for social science; UOG: University of Gondar; VCT: Voluntary counseling and testing.

## Competing interests

The authors would like to declare that we have no competing interest.

## Authors’ contributions

EE has conceived of the study, carried out the overall design and execution of the study, performed data collection and statistical analysis. AW has critically revised the design of the study, data collection techniques and helped the statistical analysis. GT has drafted the manuscript and participated in the revision of the paper. All authors read and finally approved this manuscript for submission.
